# From bites to barcodes: uncovering the hidden diversity of black flies (Diptera: Simuliidae) in Vietnam

**DOI:** 10.1186/s13071-023-05892-0

**Published:** 2023-08-07

**Authors:** Qi Yan Putt, Zubaidah Ya’cob, Peter H. Adler, Chee Dhang Chen, Yan Xin Hew, Noor Izwan-Anas, Koon Weng Lau, Mohd Sofian-Azirun, Xuan Da Pham, Hiroyuki Takaoka, Van Lun Low

**Affiliations:** 1 Tropical Infectious Diseases Research and Education Centre (TIDREC), Universiti Malaya, Kuala Lumpur, Malaysia; 2 Institute for Advanced Studies, Universiti Malaya, Kuala Lumpur, Malaysia; 3 Department of Plant and Environmental Sciences, Clemson University, Clemson, SC USA; 4 Institute of Biological Sciences, Universiti Malaya, Kuala Lumpur, Malaysia; 5 Research Center for Genetics and Reproductive Health, School of Medicine, Vietnam National University, Ho Chi Minh City, Vietnam

**Keywords:** *Simulium*, DNA barcoding, COI gene, Big zinc finger gene

## Abstract

**Background:**

Prompt and precise identification of black flies (Simuliidae) is crucial, given their biting behaviour and significant impact on human and animal health. To address the challenges presented by morphology and chromosomes in black fly taxonomy, along with the limited availability of molecular data pertaining to the black fly fauna in Vietnam, this study employed DNA-based approaches. Specifically, we used mitochondrial and nuclear-encoded genes to distinguish nominal species of black flies in Vietnam.

**Methods:**

In this study, 135 mitochondrial cytochrome c oxidase subunit I (COI) sequences were established for 45 species in the genus *Simulium* in Vietnam, encompassing three subgenera (*Gomphostilbia*, *Nevermannia*, and *Simulium*), with 64 paratypes of 27 species and 16 topotypes of six species. Of these COI sequences, 71, representing 27 species, are reported for the first time.

**Results:**

Combined with GenBank sequences of specimens from Malaysia, Myanmar, Thailand, and Vietnam, a total of 234 DNA barcodes of 53 nominal species resulted in a 71% success rate for species identification. Species from the non-monophyletic *Simulium asakoae, S. feuerborni*, *S. multistriatum*, *S. striatum*, *S. tuberosum*, and *S. variegatum* species groups were associated with ambiguous or incorrect identifications. Pairwise distances, phylogenetics, and species delimitation analyses revealed a high level of cryptic diversity, with discovery of 15 cryptic taxa. The current study also revealed the limited utility of a fast-evolving nuclear gene, big zinc finger (BZF), in discriminating closely related, morphologically similar nominal species of the *S. asakoae* species group.

**Conclusion:**

This study represents the first comprehensive molecular genetic analysis of the black fly fauna in Vietnam to our knowledge, providing a foundation for future research. DNA barcoding exhibits varying levels of differentiating efficiency across species groups but is valuable in the discovery of cryptic diversity.

**Graphical abstract:**

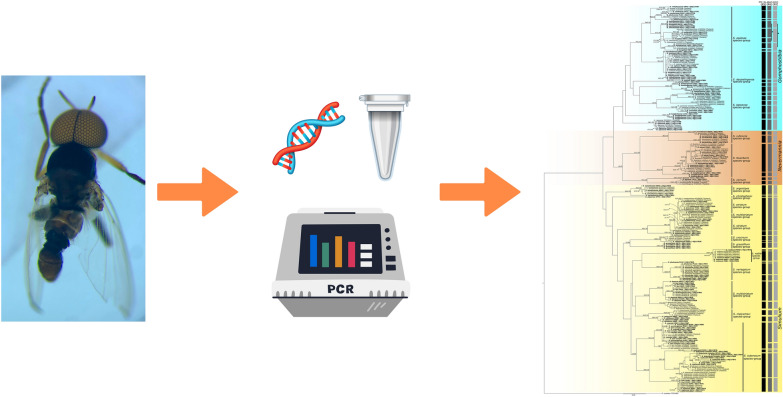

**Supplementary Information:**

The online version contains supplementary material available at 10.1186/s13071-023-05892-0.

## Background

Black flies are notorious biting insects of medical and veterinary importance, owing to their blood-feeding on humans, domesticated animals, and wildlife. Some species also act as vectors for various pathogens and parasites, including protozoa, viruses, and filarial nematodes, which can impair the health of humans and animals [1]. To date, nearly 2400 extant species of black flies have been reported worldwide [2]. In Vietnam, the diversity of these flies is striking, with 73 species found in five of the 58 surveyed provinces, 48 species of which were described in the last decade [3–10]. All known black flies in Vietnam are assigned to the genus *Simulium* and are classified in four subgenera: *Gomphostilbia* (26 species), *Montisimulium* (1 species), *Nevermannia* (8 species), and *Simulium* (38 species). Although the biting behaviour of black flies in Vietnam is little explored, a previous study in northern Vietnam found five species (*Simulium asakoae*, *S. vietnamense*, *S. chungi*, *S. grossifilum*, and *S. maenoi*) that are attracted to humans [3]. Five biting species of humans in Thailand (*Simulium asakoae*, *S. nigrogilvum, S. nodosum, S. chamlongi*, and the *S. doipuiense* complex) [11, 12], the first three of which transmit zoonotic *Onchocerca* spp. [13–16], are also present in Vietnam. *Simulium asakoae* and *S. chumpornense* are possible vectors of *Leucocytozoon* and *Trypanosoma* in domestic chickens and other birds in Thailand [17–19]. Further investigations are needed to reveal the actual biting habits of Vietnamese black flies, their associated blood-borne pathogens, and the transmission risk of zoonotic agents. As the first step, an accurate and rapid platform for the identification of black flies is vital and technically significant.

Previous studies of black flies in Vietnam have relied primarily on morphotaxonomy, contributing 43 of the 48 new nominal species in the past decade [3–10], two of which had their species status supported through molecular evidence [8, 9]. A cytotaxonomic study of the *Simulium tuberosum* species group in Vietnam revealed 15 cytoforms among six nominal species, of which five cytoforms were detected in the *S. doipuiense* complex and four cytoforms in the *S. tani* complex [20]. Some of these 15 cytoforms were later formally described based on integrated morphological, cytogenetic, and molecular approaches, namely *Simulium fuscicoxae* (*S. doipuiense* cytoform C), *S. lowi* (*S. brevipar* cytoform B), *S. rosliramlii* (*S. rufibasis* cytoform B), *S. sapaense* (*S. yuphae* cytoform B), and *S. suoivangense* (*S. tani* morphoform b or cytoform M) [2, 7, 20, 21]. Despite the significance of both approaches for species discovery and identification, the efficacy of morphotaxonomy is impeded by the presence of structural homogeneity, and cytotaxonomy is typically used only for the larvae [1]. On the other hand, molecular approaches, particularly DNA barcoding, could complement both approaches by associating unknown life stages [22, 23], revealing cryptic species [24–26] and distinguishing molecular lineages of larvae used in cytogenetic study based on DNA barcodes from adults [27]. Several studies demonstrate the effectiveness of DNA barcoding for distinguishing species previously established by morphology and cytogenetics [26, 28, 29].

The *S. asakoae* species group, as defined in [30], includes the most members (13) of any species group in Vietnam [7]. The group includes species of medical and veterinary importance. Females of most members of this group are structurally similar, rendering morphological identification difficult [3, 6, 7, 31], particularly when only females are collected from the field [32]. Previous studies show that species boundaries between certain members of the *S. asakoae* species group in Malaysia and Thailand are poorly resolved using the COI gene alone [33, 34], suggesting the need for a more variable genetic marker. The fast-evolving nuclear gene, big zinc finger (BZF), has been used in several black fly phylogenetic studies, with promising results for resolving closely related species in the *Simulium jenningsi* and *S. striatum* species groups [35–39]. However, its efficacy for delineating closely related species in the *S. asakoae* species group has yet to be tested.

The aim of this study was to delimit black fly species in Vietnam by using DNA barcoding and elucidating evolutionary relationships based on the mitochondrial COI gene. We also examined the ability of the nuclear BZF gene to differentiate members of the *S. asakoae* species group. A total of 135 specimens of 45 species, with 15, 7, and 23 species in the subgenera *Gomphostilbia*, *Nevermannia*, and *Simulium*, respectively, were examined. Included were 64 paratypes of 27 species and 16 specimens (topotypes) from type localities of six species, providing a significant way to connect formal names with genetic characterizations. A total of 71 COI sequences of 27 species generated in this study for the first time are deposited in the National Centre for Biotechnology Information (NCBI) GenBank.

## Methods

### Specimen collection and identification

Simuliid pupae were collected from five Vietnamese provinces: Lam Dong, Lao Cai, Nghe An, Thua Thien Hue, and Vinh Phuc (Table 1). Briefly, pupae were collected, following established methods [40, 41], with fine forceps from stream substrates and were reared to adults in small tubes, fixed in 80% ethanol, and stored at − 20 °C until molecular analysis. Reared males and females were identified to nominal species, using relevant identification keys [3–9].

**Table 1:** Specimens of 45 black fly species collected in Vietnam for molecular analysis, with corresponding collection information

Subgenus, species	Sample code	Collection locality	Coordinates	Elevation (m)	Collection date	GenBank accession no.	
						COI	BZF
*Gomphostilbia* Enderlein							
*Simulium asakoae* Takaoka & Davies, 1995	ASK1	Tam Dao, Vinh Phuc	21°25′41.2″ N, 105°36′55.7″ E	997	2013-11-09	OQ117762	OQ427828
	ASK2	Hue, Thua Thien Hue	16°10′23.392″ N, 107°43′32.357″ E	N/A	2014-02-22	OQ117763	OQ427829
	ASK3	Lac Duong, Lam Dong	11°59′26.0″ N, 108°22′06.0″ E	1443	2014-04-22	OQ117764	OQ427830
	ASK4	Quy Chau, Nghe An	19°20′12.147″ N, 105°09′15.908″ E	N/A	2015-12-11	OQ117765	OQ427831
*S. chaudinhense* Takaoka & Sofian-Azirun, 2017	CDH1^a^	Quy Chau, Nghe An	19°30′46.589″ N, 105°09′10.470″ E	80	2015-12-11	OQ117766	OQ427832
	CDH2^a^	Quy Chau, Nghe An	19°30′46.589″ N, 105°09′10.470″ E	80	2015-12-11	OQ117767	OQ427833
	CDH3^a^	Quy Chau, Nghe An	19°30′46.589″ N, 105°09′10.470″ E	80	2015-12-11	OQ117768	OQ427834
	CDH4^a^	Quy Chau, Nghe An	19°30′46.589″ N, 105°09′10.470″ E	80	2015-12-11	OQ117769	OQ427835
*S. confertum* Takaoka & Sofian-Azirun, 2015	CFT1^a^	Lac Duong, Lam Dong	12°07′59.430″ N, 108°35′42.001″ E	1492	2014-04-24	OQ117770	OQ427836
	CFT2	Sa Pa, Lao Cai	22°19′04.333″ N, 103°52′04.265″ E	1223	2014-12-21	OQ117771	OQ427837
	CFT3	Sa Pa, Lao Cai	22°19′04.333″ N, 103°52′04.265″ E	1223	2014-12-21	OQ117772	OQ427838
*S. hongthaii* Takaoka, Sofian-Azirun & Ya'cob, 2014	HGT1	Hue, Thua Thien Hue	16°10′23.392″ N, 107°43′32.357″ E	N/A	2014-02-22	OQ117773	OQ427839
	HGT2^a^	Tam Dao National Park, Vinh Phuc	21°27′30.1" N, 105°38′56.5″ E	997	2013-11-08	OQ117774	OQ427840
	HGT3	Bach Ma National Park, Thua Thien Hue	16°13′56.211″ N, 107°51′18.671″ E	432	2014-02-23	OQ117775	OQ427841
	HGT4	Sa Pa, Lao Cai	22°19′20.0" N, 103°50′55.0″ E	1019	2014-12-21	OQ117776	OQ427842
*S. phulocense* Takaoka & Chen, 2015	PLO1^b^	Bach Ma National Park, Thua Thien Hue	16°11′41.099″ N, 107°51′47.675″ E	1186	2014-02-23	OQ117777	N/A
	PLO2^b^	Bach Ma National Park, Thua Thien Hue	16°11′41.099″ N, 107°51′47.675″ E	1186	2014-02-23	OQ117778	OQ427843
	PLO3^a^	Bach Ma National Park, Thua Thien Hue	16°11′45.123″ N, 107°50′55.6″ E	1186	2014-02-23	OQ117779	OQ427844
*S. sanchayense* Takaoka & Lau, 2017	SCY1^b^	Tam Dao National Park, Vinh Phuc	21°27′30.1″ N, 105°38′56.5″ E	975	2013-11-08	OQ117780	OQ427845
	SCY2^b^	Tam Dao National Park, Vinh Phuc	21°27′30.1″ N, 105°38′56.5" E	975	2013-11-08	OQ117781	OQ427846
	SCY3^a^	Tam Dao National Park, Vinh Phuc	21°27′30.1″ N, 105°38′56.5" E	975	2013-11-08	OQ117782	OQ427847
	SCY4^a^	Tam Dao National Park, Vinh Phuc	21°27′30.1" N, 105°38′56.5" E	975	2013-11-08	OQ117783	OQ427848
*S. tamdaoense* Takaoka, Sofian-Azirun & Ya’cob, 2014	TAM1	Pu Mat National Park, Nghe An	N/A	N/A	2015-12-11	OQ117784	OQ427849
*S. vinhphucense* Takaoka & Low, 2017	VPH1	Pu Mat National Park, Nghe An	N/A	N/A	2015-12-11	OQ117785	OQ427850
	VPH2	Pu Mat National Park, Nghe An	N/A	N/A	2015-12-11	OQ117786	N/A
	VPH3^a^	Tam Dao National Park, Vinh Phuc	21°27′30.1" N, 105°38′56.5" E	975	2013-11-08	OQ117787	OQ427851
*S. dachaisense* Takaoka & Lau, 2015	DCS1^a^	Lac Duong, Lam Dong	12°08′32.409″ N, 108°38′58.318″ E	1440	2014-04-24	OQ117802	N/A
	DCS2^a^	Lac Duong, Lam Dong	12°08′32.409″ N, 108°38′58.318″ E	1440	2014-04-24	OQ117803	N/A
*S. lamdongense* Takaoka & Sofian-Azirun, 2015	LDG1	Pu Mat, Nghe An	N/A	N/A	2015-12-09	OQ117792	N/A
	LDG2	Pu Mat, Nghe An	N/A	N/A	2015-12-09	OQ117793	N/A
	LDG3^b^	Lac Duong, Lam Dong	12°01′24.991″ N, 108°25′30.704″ E	1539	2014-04-25	OQ117794	N/A
	LDG4^a^	Lac Duong, Lam Dong	12°01′24.991″ N, 108°25′30.704″ E	1539	2014-04-25	OQ117795	N/A
*S. longlanhense* Takaoka & Ya’cob, 2015	LLH1^a^	Lac Duong, Lam Dong	12°10′56.408″ N, 108°40′48.152″ E	1452	2014-04-24	OQ117796	N/A
	LLH2^a^	Lac Duong, Lam Dong	12°10′56.408″ N, 108°40′48.152″ E	1452	2014-04-24	OQ117797	N/A
	LLH3^a^	Lac Duong, Lam Dong	12°10′56.408″ N, 108°40′48.152″ E	1452	2014-04-24	OQ117798	N/A
	LLH4^a^	Lac Duong, Lam Dong	12°10′56.408″ N, 108°40′48.152″ E	1452	2014-04-24	OQ117799	N/A
*S. ngaoense* Takaoka, Srisuka, & Saeung, 2018	NGA1	Quy Chau, Nghe An	19°30′46.9″ N, 105°09′10.5″ E	80	2015-12-11	OQ117788	N/A
	NGA2	Quy Chau, Nghe An	19°30′46.9″ N, 105°09′10.5″ E	80	2015-12-11	OQ117789	N/A
	NGA3	Quy Chau, Nghe An	19°30′45.607″ N, 105°09′16.430″ E	80	2015-12-11	OQ117790	N/A
	NGA4	Quy Chau, Nghe An	19°30′45.607″ N, 105°09′16.430″ E	80	2015-12-11	OQ117791	N/A
*S. yvonneae* Takaoka & Low, 2019	YVN1^a^	Hugen Anh Son, Nghe An	18°56′41.456″ N, 105°02′16.396″ E	16–162	2015-12-10	OQ117804	N/A
	YVN2^a^	Hugen Anh Son, Nghe An	18°56′41.456″ N, 105°02′16.396″ E	16–162	2015-12-10	OQ117805	N/A
	YVN3^a^	Hugen Anh Son, Nghe An	18°56′41.456″ N, 105°02′16.396″ E	16–162	2015-12-10	OQ117806	N/A
*S. eshimai* Takaoka & Adler, 2017	ESM1^a^	Sa Pa, Lao Cai	22°22′22.719″ N, 103°45′24.852″ E	1728	2014-12-20	OQ117800	N/A
	ESM2^a^	Sa Pa, Lao Cai	22°22′22.719″ N, 103°45′24.852″ E	1728	2014-12-20	OQ117801	N/A
*Nevermannia* Enderlein							
*S. bachmaense* Takaoka, Sofian-Azirun & Ya’cob, 2014	BMA1^a^	Bach Ma National Park, Thua Thien Hue	16°11′41.099″ N, 107°51′47.675″ E	1414	2014-02-23	OQ117807	N/A
*S. langbiangense* Sofian-Azirun & Ya’cob, 2014	LBG1^a^	Lac Duong, Lam Dong	100 m left from the stream of the type locality of *S. phami*	1540	2014-04-25	OQ117808	N/A
	LBG2^a^	Lac Duong, Lam Dong	100 m left from the stream of the type locality of *S. phami*	1540	2014-04-25	OQ117809	N/A
	LBG3^a^	Lac Duong, Lam Dong	100 m left from the stream of the type locality of *S. phami*	1540	2014-04-25	OQ117810	N/A
*S. maeaiense* Takaoka & Srisuka, 2011	MAE1	Sa Pa, Lao Cai	22°21′28.378″ N, 103°45′52.084″ E	1893	2014-12-20	OQ117811	N/A
	MAE2	Sa Pa, Lao Cai	22°21′28.378″ N, 103°45′52.084″ E	1893	2014-12-20	OQ117812	N/A
*S. phami* Takaoka, Sofian-Azirun & Ya’cob, 2014	PHM1^a^	Lac Duong, Lam Dong	12°01′24.991″ N, 108°25′30.704″ E	1539	2014-04-25	OQ117813	N/A
	PHM2^a^	Lac Duong, Lam Dong	12°01′24.991″ N, 108°25′30.704″ E	1539	2014-04-25	OQ117814	N/A
*S. pumatense* Takaoka, Low & Pham, 2019	PMT1^a^	Pu Mat National Park, Nghe An	18˚58′03.270″ N, 104˚48′07.811″ E	223	2015-12-09	OQ117815	N/A
	PMT2^a^	Pu Mat National Park, Nghe An	18˚58′03.270″ N, 104˚48′07.811″ E	223	2015-12-09	OQ117816	N/A
*S. aureohirtum* Brunetti, 1911	ARH1	Quy Chau, Nghe An	N/A	N/A	2015-12-10	OQ117817	N/A
	ARH2	Quy Chau, Nghe An	19°20′12.147″ N, 105°09′15.908″ E	80	2015-12-11	OQ117818	N/A
	ARH3	Lac Duong, Lam Dong	11°59′25.511″ N, 108°22′06.350″ E	1443	2014-04-22	OQ117819	N/A
	ARH4	Lac Duong, Lam Dong	11°59′25.511″ N, 108°22′06.350″ E	1443	2014-04-22	OQ117820	N/A
*S. tayense* Takaoka & Ya’cob, 2017	TAY1^a^	Sa Pa, Lao Cai Province	22°22′22.719″ N, 103°45′24.852″ E	1728	2014-12-20	OQ117821	N/A
	TAY2^a^	Sa Pa, Lao Cai Province	22°22′22.719″ N, 103°45′24.852″ E	1728	2014-12-20	OQ117822	N/A
*Simulium* Latreille							
*S. sansahoense* Takaoka & Chen, 2017	SSH1^a^	Sa Pa, Lao Cai Province	22°19′44.349″ N, 103°49′49.930″ E	1194	2014-12-20	OQ117823	N/A
	SSH2^a^	Sa Pa, Lao Cai Province	22°19′44.349″ N, 103°49′49.930″ E	1194	2014-12-20	OQ117824	N/A
	SSH3^a^	Sa Pa, Lao Cai Province	22°19′44.349″ N, 103°49′49.930″ E	1194	2014-12-20	OQ117825	N/A
	SSH4^a^	Sa Pa, Lao Cai Province	22°19′44.349″ N, 103°49′49.930″ E	1194	2014-12-20	OQ117826	N/A
*S. atipornae* Takaoka, Srisuka & Choochote, 2014	ATP1	Bach Ma National Park, Thua Thien Hue	16°11′42.061″ N, 107°51′27.955″ E	1273	2014-02-23	OQ117827	N/A
*S. vietnamense* Takaoka, Sofian-Azirun & Chen, 2014	VNM1^a^	Tam Dao National Park, Vinh Phuc	21°20′08.1″ N, 105°30′32.4″ E	997	2013-11-09	OQ117828	N/A
*S. grossifilum* Takaoka & Davies, 1995	GSF1	Tam Dao National Park, Vinh Phuc	21°20′08.1″ N, 105°30′32.4″ E	997	2013-11-09	OQ117829	N/A
	GSF2	Tam Dao National Park, Vinh Phuc	21°20′08.1″ N, 105°30′32.4″ E	997	2013-11-09	OQ117830	N/A
	GSF3	Tam Dao National Park, Vinh Phuc	21°20′08.1″ N, 105°30′32.4″ E	997	2013-11-09	OQ117831	N/A
	GSF4	Tam Dao National Park, Vinh Phuc	21°20′08.1″ N, 105°30′32.4″ E	997	2013-11-09	OQ117832	N/A
*S. obliquum* Takaoka & Low, 2017	OBQ1^a^	Pu Mat National Park, Nghe An	18°58′45.918″ N, 109°50′10.693″ E	162	2015-12-09	OQ117833	N/A
	OBQ2^a^	Pu Mat National Park, Nghe An	18°58′45.918″ N, 109°50′10.693″ E	162	2015-12-09	OQ117834	N/A
	OBQ3^a^	Pu Mat National Park, Nghe An	18°58′45.918″ N, 109°50′10.693″ E	162	2015-12-09	OQ117835	N/A
	OBQ4^a^	Pu Mat National Park, Nghe An	18°58′45.918″ N, 109°50′10.693″ E	162	2015-12-09	OQ117836	N/A
*S. daoense* Takaoka & Adler, 2017	DAO1^a^	Sa Pa, Lao Cai	22°23′03.208″ N, 103°50′58.990″ E	1315	2014-12-22	OQ117837	N/A
	DAO2^a^	Sa Pa, Lao Cai	22°23′03.208″ N, 103°50′58.990″ E	1315	2014-12-22	OQ117838	N/A
	DAO3^a^	Sa Pa, Lao Cai	22°23′03.208″ N, 103°50′58.990″ E	1315	2014-12-22	OQ117839	N/A
	DAO4^a^	Sa Pa, Lao Cai	22°23′03.208″ N, 103°50′58.990″ E	1315	2014-12-22	OQ117840	N/A
*S. lacduongense* Takaoka & Ya′cob, 2015	LAC1^a^	Lac Duong, Lam Dong	12°08′32.409″ N, 108°38′58.318″ E	1440	2014-04-24	OQ117841	N/A
*S. laui* Takaoka & Sofian-Azirun, 2015	LAU1^a^	Lac Duong, Lam Dong	12°01′49.981″ N, 108°21′51.175″ E	1415	2014-04-23	OQ117842	N/A
	LAU2^a^	Lac Duong, Lam Dong	12°01′49.981″ N, 108°21′51.175″ E	1415	2014-04-23	OQ117843	N/A
	LAU3^a^	Lac Duong, Lam Dong	12°01′49.981″ N, 108°21′51.175″ E	1415	2014-04-23	OQ117844	N/A
	LAU4^a^	Lac Duong, Lam Dong	12°01′49.981″ N, 108°21′51.175″ E	1415	2014-04-23	OQ117845	N/A
*S. nodosum* Puri, 1933	NDS1	Quy Chau, Nghe An	N/A	N/A	2015-12-10	OQ117846	N/A
	NDS2	Quy Chau, Nghe An	19°30′45.487″ N, 105°09′09.261″ E	73	2015-12-11	OQ117847	N/A
	NDS3	Lac Duong, Lam Dong	12°08′32.409″ N, 108°38′58.318″ E	1439	2014-04-24	OQ117849	N/A
	NDS4	Tam Dao, Vinh Phuc	21°27′40.4" N, 105°34′20.0" E	283	2013-11-11	OQ117848	N/A
*S. nakhonense* Takaoka & Suzuki, 1984	NKH1	Pu Mat, Nghe An	18°58′45.918″ N, 109°50′10.693″ E	162	2015-12-09	OQ117850	OQ427852
	NKH2	Pu Mat, Nghe An	N/A	N/A	2015-12-10	OQ117851	N/A
	NKH3	Pu Mat, Nghe An	N/A	N/A	2015-12-10	OQ117852	N/A
	NKH4	Pu Mat, Nghe An	N/A	N/A	2015-12-10	OQ117853	N/A
*S. quinquestriatum* Shiraki, 1935	QQS1	Quy Chau, Nghe An	19°30′45.607″ N, 105°09′16.430″	80	2015-12-11	OQ117854	N/A
*S. tavanense* Takaoka & Sofian-Azirun, 2017	TVA1^a^	Sa Pa, Lao Cai	22°18′33.255″ N, 103°53′12.129″ E	999	2014-12-21	OQ117855	N/A
	TVA2^a^	Sa Pa, Lao Cai	22°18′23.788″ N, 103°53′42.780″ E	999	2014-12-21	OQ117856	N/A
	TVA3^a^	Sa Pa, Lao Cai	22°18′23.788″ N, 103°53′42.780″ E	999	2014-12-21	OQ117857	N/A
*S. taythienense* Takaoka, Sofian-Azirun & Ya’cob, 2014	TTY1	Pu Mat, Nghe An	N/A	N/A	2015-12-09	OQ117858	N/A
	TTY2	Pu Mat, Nghe An	N/A	N/A	2015-12-09	OQ117859	N/A
	TTY3	Pu Mat, Nghe An	N/A	N/A	2015-12-09	OQ117860	N/A
	TTY4	Pu Mat, Nghe An	N/A	N/A	2015-12-09	OQ117861	N/A
*S. xuandai* Takaoka, Sofian-Azirun & Ya'cob, 2014	XDA1	Da Lat, Lam Dong	11°59′26.0″ N, 108°22′06.0″ E	1443	2014-04-22	OQ117862	N/A
*S. congi* Takaoka & Sofian-Azirun, 2015	COG1^a^	Da Lat, Lam Dong	12°06′06.888″ N, 108°22′02.797″ E	1722	2014-04-23	OQ117863	N/A
*S. doipuiense* Takaoka & Choochote, 2005 complex	DPU1	Sa Pa, Lao Cai	22°18′48.0" N, 103°53′10.0" E	1105	2014-12-21	OQ117864	N/A
	DPU2	Sa Pa, Lao Cai	22°18′48.0" N, 103°53′10.0" E	1105	2014-12-21	OQ117865	N/A
	DPU3	Sa Pa, Lao Cai	22°15′30.0" N, 103°50′39.0" E	1201	2014-12-22	OQ117866	N/A
	DPU4	Sa Pa, Lao Cai	22°23′03.208″ N, 103°50′58.990″ E	N/A	2014-04-23	OQ117867	N/A
*S. lowi* Takaoka & Adler, 2017	LOW1^a^	Tam Dao, Vinh Phuc	21°27′30.1" N, 105°38′56.5" E	975	2013-11-08	OQ117868	N/A
	LOW2^a^	Tam Dao, Vinh Phuc	21°27′30.1" N, 105°38′56.5" E	975	2013-11-08	OQ117869	N/A
*S. rosliramlii* Takaoka & Chen, 2017	ROS1^b^	Sa Pa, Lao Cai	22°22′22.719″ N, 103°45′24.852″ E	1728	2014-12-20	OQ117870	N/A
	ROS2^b^	Sa Pa, Lao Cai	22°22′22.719″ N, 103°45′24.852″ E	1728	2014-12-20	OQ117871	N/A
	ROS3^b^	Sa Pa, Lao Cai	22°22′22.719″ N, 103°45′24.852″ E	1728	2014-12-20	OQ117872	N/A
	ROS4^b^	Sa Pa, Lao Cai	22°22′22.719″ N, 103°45′24.852″ E	1728	2014-12-20	OQ117873	N/A
*S. sapaense* Takaoka & Low, 2017	SAP1^a^	Sa Pa, Lao Cai	22°22′05.320″ N, 103°47′34.403″ E	1680	2014-12-20	OQ117874	N/A
	SAP2^a^	Sa Pa, Lao Cai	22°21′43.110″ N, 103°47′19.221″ E	1750	2014-12-20	OQ117875	N/A
	SAP3^a^	Sa Pa, Lao Cai	22°23′03.208″ N, 103°50′58.990″ E	1750	2014-12-22	OQ117876	N/A
*S. suoivangense* Takaoka & Pham, 2017	SVG1^b^	Lac Duong, Lam Dong	12°10′56.408″ N, 108°40′48.152″ E	1452	2014-04-22	OQ117877	N/A
	SVG2^b^	Lac Duong, Lam Dong	12°10′56.408″ N, 108°40′48.152″ E	1452	2014-04-22	OQ117878	N/A
	SVG3^b^	Lac Duong, Lam Dong	12°10′56.408″ N, 108°40′48.152″ E	1452	2014-04-22	OQ117879	N/A
	SVG4^a^	Lac Duong, Lam Dong	12°10′56.408″ N, 108°40′48.152″ E	1452	2014-04-22	OQ117880	N/A
*S. tani* Takaoka & Davies, 1995 complex	TN1	Lac Duong, Lam Dong	12°01′24.991″ N, 108°25′30.704″ E	1539	2014-04-25	OQ117881	N/A
	TN2	Luoi, Thua Thien Hue	16°18′16.209″ N, 107°12′48.027″ E	628	2014-02-24	OQ117882	N/A
	TN3	Quy Chau, Nghe An	18°58′45.918″ N, 109°50′10.693″ E	162	2015-12-09	OQ117883	N/A
	TN4	Quy Chau, Nghe An	19°30′45.487″ N, 105°09′09.261″ E	73	2015-12-11	OQ117884	N/A
*S. xuandei* Takaoka & Pham, 2015	XDE1^a^	Lac Duong, Lam Dong	12°07′59.430″ N, 108°35′42.001″ E	1492	2014-04-24	OQ117885	N/A
	XDE2^a^	Lac Duong, Lam Dong	12°07′59.430″ N, 108°35′42.001″ E	1492	2014-04-24	OQ117886	N/A
	XDE3^a^	Lac Duong, Lam Dong	12°07′59.430″ N, 108°35′42.001″ E	1492	2014-04-24	OQ117887	N/A
	XDE4^a^	Lac Duong, Lam Dong	12°07′59.430″ N, 108°35′42.001″ E	1492	2014-04-24	OQ117888	N/A
*S. chamlongi* Takaoka & Suzuki, 1984	CAM1	Lac Duong, Lam Dong	12°07′59.430″ N, 108°35′42.001″ E	1492	2014-04-24	OQ117889	N/A
	CAM2	Lac Duong, Lam Dong	12°07′59.430″ N, 108°35′42.001″ E	1492	2014-04-24	OQ117890	N/A
	CAM3	Lac Duong, Lam Dong	12°07′59.430″ N, 108°35′42.001″ E	1492	2014-04-24	OQ117891	N/A
	CAM4	Lac Duong, Lam Dong	12°07′59.430″ N, 108°35′42.001″ E	1492	2014-04-24	OQ117892	N/A
*S. phuluense* Takaoka & Sofian-Azirun, 2017	PLU1^b^	Sa Pa, Lao Cai	22°21′43.110″ N, 103°47′19.221″ E	1750	2014-12-20	OQ117893	N/A
	PLU2^b^	Sa Pa, Lao Cai	22°19′44.349″ N, 103°49′49.930″ E	1194	2014-12-20	OQ117894	N/A
	PLU3^b^	Sa Pa, Lao Cai	22°23′03.208″ N, 103°50′58.990″ E	1192	2014-12-23	OQ117895	N/A
	PLU4^b^	Sa Pa, Lao Cai	22°23′03.208″ N, 103°50′58.990″ E	1192	2014-12-23	OQ117896	N/A

^a^Paratype, ^b^topotype

### DNA extraction, amplification, and sequencing

Genomic DNA was extracted from the thorax(es) or leg(s) of one to four reared adults per species using the NucleoSpin Tissue DNA Extraction Kit (Macherey-Nagel, Germany). The COI gene was amplified using the DNA barcoding primers, LCO1490 and HCO2198 [42]. The cycling parameters were based on those described by [26]. Amplification of the BZF gene involved 26 individuals representing eight species of the *Simulium asakoae* species group from Vietnam (*S. asakoae* s. str., *S. chaudinhense*, *S. confertum*, *S. hongthaii*, *S. phulocense*, *S. sanchayense*, *S. tamdaoense*, and *S. vinhphucense*). For the BZF gene, an approximately 440-bp fragment was amplified using our newly designed primers: 2nd_BZF_F (5′-GAAAACGAGGACACCGAAGA-3′) and 2nd_BZF_R (5′-CCCATCTTTGCACTGTTTGC-3′). The cycling conditions included an initial denaturation at 94 °C for 2 min; 37 cycles at 94 °C for 30 s (denaturation), 53–55 °C for 45 s (annealing), and 72 °C for 45 s (elongation); and a final elongation at 72 °C for 4 min. Some individuals that were not successfully amplified using the primers 2nd_BZF_F and 2nd_BZF_R, were subjected to nested polymerase chain reaction (PCR) amplification. The first PCR used the primer pairs (BBZF_F and BBZF_R) of [39]. For the second PCR, 1 µl of the product from the first amplification was subsequently amplified using the primer pairs (2nd_BZF_F and 2nd_BZF_R) from the present study. The reaction conditions for the nested PCR followed those of the PCR for amplifying the BZF gene, except the annealing phase was 47–55 °C for 45 s. Consequently, 24 of the 26 individuals were successfully amplified by the BZF gene. Amplifications of the COI and BZF gene were performed in a final reaction volume of 25 µl, containing 1 µl of genomic DNA of black flies, 12.5 µl of MyTaq Red Mix (BioLine, UK), and 1 µl of each forward and reverse primer (final concentration of both primers: 400 nM). The PCR products were checked by electrophoresis in 1.5% agarose gel pre-stained with SYBR Safe DNA gel stain (Invitrogen, USA). Successfully amplified products were purified and sequenced by Apical Scientific (Malaysia).

### Data analyses

In total, 135 COI sequences representing 45 species and 24 BZF sequences representing eight species of the *S. asakoae* species group in Vietnam were deposited in the NCBI GenBank under accession numbers OQ117762–OQ117896 and OQ427828–OQ427851, respectively. Relevant information on these 45 species, with respective COI sequences, are also deposited in the Global Biodiversity Information Facility (GBIF), an open access database. For comparison, 107 COI sequences of black flies from Malaysia, Myanmar, Vietnam, and Thailand were retrieved from the NCBI GenBank database as reference sequences, for a final COI dataset of 242 sequences for subsequent DNA barcode analyses. All COI and BZF sequences were aligned in MAFFT v7 [43] and trimmed using BioEdit v7. 2. 5 [44]. A partition homogeneity test using Paup* 4.0b10 [45] was performed with 1000 replicates to evaluate statistical congruence between the COI and BZF gene fragments in determining whether both fragments could be combined. The partition homogeneity test generated a *p*-value of 0.001, indicating individual partitions were incongruent. Despite a low *p*-value, incongruent datasets can be combined to improve phylogenetic accuracy [46]. Hence, the BZF sequences were concatenated with the corresponding COI sequences for the subsequent data analyses.

For COI and concatenated datasets, pairwise genetic distances within and among species were estimated based on an uncorrected *p*-distance with 1000 bootstrap replicates implemented in Mega 11 [47]. For the COI dataset, success rates of the DNA barcodes in species identification were assessed based on “Best Match” (BM) and “Best Close Match” (BCM) criteria using TaxonDNA [48]. Under the “Best Match” criteria, correct identification is indicated when the query sequence is matched to the sequences with the smallest genetic distance that are all conspecific [48]. The assignation is similar in the “Best Close Match” criteria, except this smallest genetic distance is within a threshold distance that is below the 95th percentile of all intraspecific genetic distances found [48]. This threshold (2.76%) is estimated ad hoc for the COI dataset [49]. Ambiguous and incorrect identifications were rendered when species with single COI barcodes were used as queries, as there are no other conspecific reference sequences in the dataset to allow these single COI barcodes to be matched [50]. Therefore, eight species (*Simulium tamdaoense*, *S. bachmaense*, *S. atipornae*, *S. vietnamense*, *S. lacduongense*, *S. quinquestriatum*, *S. xuandai*, and *S. congi*), each with single COI sequences removed, left 234 COI sequences to be subjected to the "Best Match" and "Best Close Match" analyses.

Phylogenetic analyses were performed using Bayesian inference (BI) and maximum likelihood (ML) analyses for both datasets. Prior to performing phylogenetic analyses, Kakusan4 [51] was used to determine the best-fit nucleotide substitution model. BI analysis was performed using MrBayes v3.2.7a [52] implemented in CIPRES Science Gateway v3.3 online portal (https://www.phylo.org/) [53]. The general time reversible substitution model with a gamma shape parameter of 0.719 and a proportion of invariable sites of 0.487 (GTR + G + I) was preferred for the COI dataset. In contrast, the Hasegawa-Kishono-Yano substitution model with a gamma shape parameter of 0.212 (HKY + G) was favoured for the concatenated dataset. The posterior probability distributions of the BI trees were inferred from two independent runs of 25 and 5 million generations, respectively, with four Markov chain Monte Carlo (MCMC) chains until the average standard deviation of split frequencies reached < 0.01, with tree sampling every 100 generations and a relative burn-in of 25%. ML analysis was performed using the RAxML webserver (https://raxml-ng.vital-it.ch/#/) [54] based on 100 bootstrap replicates. The GTR substitution model with a gamma shape parameter of 0.657 and a proportion of invariable sites of 0.459 (GTR + G + I) was preferred for the COI dataset. The GTR substitution model with a gamma shape parameter of 0.202 (GTR + G) was favoured for the concatenated dataset. *Parasimulium crosskeyi* was used as the outgroup in the COI dataset, whereas *Simulium nakhonense* and *S. reptans* were the outgroups in the concatenated dataset.

For COI and concatenated datasets, species delimitation analyses were performed using Poisson Tree Processes (PTP) [55], Assemble Species by Automatic Partitioning (ASAP) [56], and General Mixed Yule Coalescent (GMYC) [57, 58] to estimate the number of operational taxonomic units (OTUs). The PTP analysis was conducted in the bPTP webserver (https://species.h-its.org/ptp/). By using the ML tree generated from the RAxML analysis as the input tree, the PTP analysis was run for 500,000 and 100,000 MCMC generations for the COI and concatenated datasets, respectively, with the other settings set as default. For the COI dataset, delimitation results were based on the maximum likelihood partition (PTP_ML), whereas for the concatenated dataset, delimitation results were based on the Bayesian partition (bPTP). The ASAP analysis was run online (https://bioinfo.mnhn.fr/abi/public/asap/) under default parameters. For the COI dataset, delimitation results were based on the partition that ranked eighth, with an ASAP score of 10.5, where the threshold distances were more relevant to the current dataset than those of other partitions despite the poorer ASAP score than other partitions [56]. For the concatenated dataset, delimitation results were based on the best partition with an ASAP score of 2.0. To commence the GMYC analysis for the COI dataset, an ultrametric tree was obtained using BEAST v2.6.6 [59] run on CIPRES Science Gateway v3.3 online portal (https://www.phylo.org/) [53]. An XML input file was generated using BEAUti v2.6.6 [59] with the best-fitting model, the GTR substitution model with a gamma shape parameter of 0.585, and a proportion of invariable sites of 0.488 (GTR + G + I), determined by jModelTest2 [60] via CIPRES Science Gateway v3.3 online portal (https://www.phylo.org/) [53]. Under a strict molecular clock and the Yule speciation model with other parameters in the priors remaining as default, the dataset was run for 40 million generations, sampling every 1000 generations. Steps to implement the GMYC analysis for the concatenated datasets were similar to those of the COI dataset, except the best-fit model adopted was the HKY substitution model with a gamma shape parameter of 0.521 and a proportion of invariable sites of 0.474 (HKY + G + I), and the BEAST analysis was run with one million generations. Tracer v1.7.2 [61] was used to examine effective sample sizes (ESS) of all parameters that exceeded 200. The output trees generated by BEAST v2.6.6 were summarized in TreeAnnotator v2.6.6 [59] to construct a maximum clade credibility tree, with a 10% burn-in, 0.0 posterior probability, and median node heights. The GMYC analyses were performed in RStudio [62], using "ape" [63], "paran" [64], "rncl" [65], and "splits" [66] R packages.

## Results

Intraspecific genetic divergence for the 61 morphospecies ranged from 0.00 to 15.10%, with the highest value of maximum variation (15.10%) in *Simulium daoense* (Table 2). Interspecific genetic divergence for the 61 nominal species ranged from 0.00 to 18.56%, with the lowest value of minimum interspecific genetic variation (0.00%) between some members in the *Simulium asakoae*, *S. multistriatum*, *S. tuberosum*, and *S. variegatum* species groups (Additional file 1: Table S1; see Additional file 2: Table S2 for intra- and interspecific genetic distances for members of the *S. asakoae* species group based on concatenated COI+BZF sequences).

**Table 2:** List of 61 black fly species, number of specimens with COI sequences (*n*) and ranges followed by means of intraspecific genetic distances based on uncorrected *p*-distance expressed as percentages; the 45 species in Vietnam are in bold

Subgenus, species group	Species	*n* (current study)	Intraspecific genetic distances
*Gomphostilbia* Enderlein			
*Simulium asakoae* species group	***S. asakoae***	11**(4)**	0–1.73 (0.91)
	*S. chaowaense*	2	0.50
	***S. chaudinhense***	**(4)**	0–0.99 (0.66)
	***S. confertum***^**a**^	**(3)**	0–5.45 (3.63)
	***S. hongthaii***^**a**^	**(4)**	3.47–8.66 (6.06)
	*S. maehongsonense*	2	0.00
	*S. myanmarense*	3	0.25–0.50 (0.33)
	***S. phulocense***^**a**^	**(3)**	0.25–5.20 (3.47)
	*S. puaense*	2	0.74
	*S. rampae*	2	0.00
	***S. sanchayense***	**(4)**	0.00
	*S. songense*	2	0.25
	***S. tamdaoense***	**(1)**	–
	***S. vinhphucense***^**a**^	**(3)**	0.74–7.92 (5.28)
*S. batoense* species group	***S. dachaisense***	**(2)**	0.25
	*S. duolongum*	3	0.00
	***S. lamdongense***^**a**^	6**(4)**	0–5.45 (3.50)
	***S. longlanhense***	**(4)**	0–0.74 (0.37)
	***S. ngaoense***	7**(4)**	0–0.50 (0.26)
	*S. siamense* complex	3	0.50–1.24 (0.83)
	***S. yvonneae***^**a**^	5**(3)**	0–9.41 (5.59)
*S. darjeelingense* species group	***S. eshimai***	**(2)**	1.98
*Nevermannia* Enderlein			
*S. feuerborni* species group	***S. bachmaense***	**(1)**	–
	*S. feuerborni* cytoform A	4	0.25–0.50 (0.37)
	***S. langbiangense***	**(3)**	0–0.50 (0.33)
	***S. maeaiense***	5**(2)**	0.50–2.48 (1.91)
	***S. phami***	**(2)**	1.73
	***S. pumatense***	6**(2)**	0–2.48 (1.60)
*S. ruficorne* species group	***S. aureohirtum***^**a**^	8**(4)**	0–8.42 (4.43)
*S. vernum* species group	*S. chomthongense*	3	0.50–0.99 (0.66)
	***S. tayense***	**(2)**	0.50
*Simulium* Latreille			
*S. argentipes* species group	***S. sansahoense***^**a**^	7**(4)**	0–4.95 (1.90)
*S. christophersi* species group	***S. atipornae***	**(1)**	–
*S. crocinum* species group	*S. rudnicki*	3	0.25–0.50 (0.33)
	***S. vietnamense***	**(1)**	–
*S. grossifilum* species group	***S. grossifilum***	**(4)**	0.00
*S. malyschevi* species group	***S. obliquum***	**(4)**	0.25–1.98 (1.20)
	*S. siripoomense*	4	0.74–1.98 (1.28)
*S. multistriatum* species group	***S. daoense***^**a**^	12**(4)**	0–15.10 (7.10)
	***S. lacduongense***	**(1)**	–
	***S. laui***	**(4)**	0–0.25 (0.17)
	*S. ubonae*	4	0.00
*S. nobile* species group	***S. nodosum***	8**(4)**	0–2.97 (0.81)
*S. striatum* species group	***S. nakhonense***^**a**^	8**(4)**	0–10.15 (5.23)
	***S. quinquestriatum***	**(1)**	–
	***S. tavanense***	**(3)**	0.74–1.98 (1.49)
	***S. taythienense***	**(4)**	0.74–1.98 (1.11)
	*S. wangkwaiense*	4	0.25–1.73 (1.07)
	***S. xuandai***	**(1)**	–
*S. tuberosum* species group	***S. congi***	**(1)**	–
	***S. doipuiense* complex**^**a**^	8**(4)**	0–7.92 (3.53)
	***S. lowi***	**(2)**	0.00
	***S. rosliramlii***^**a**^	5**(4)**	1.24–8.17 (4.63)
	***S. sapaense***	**(3)**	0.25–0.50 (0.33)
	***S. suoivangense***	**(4)**	0.50–2.72 (1.94)
	***S. tani* complex**^**a**^	9**(4)**	0.50–5.94 (3.46)
	*S. tenebrosum* complex	4	0.50–1.49 (0.99)
	***S. xuandei***	**(4)**	0.74–2.23 (1.53)
	*S. yuphae*	4	0–0.25 (0.12)
*S. variegatum* species group	***S. chamlongi***^**a**^	8**(4)**	0–4.46 (2.48)
	***S. phuluense***^**a**^	**(4)**	0–4.46 (2.27)

^a^Species having taxa with intraspecific variation > 3%

**Fig. 1:**
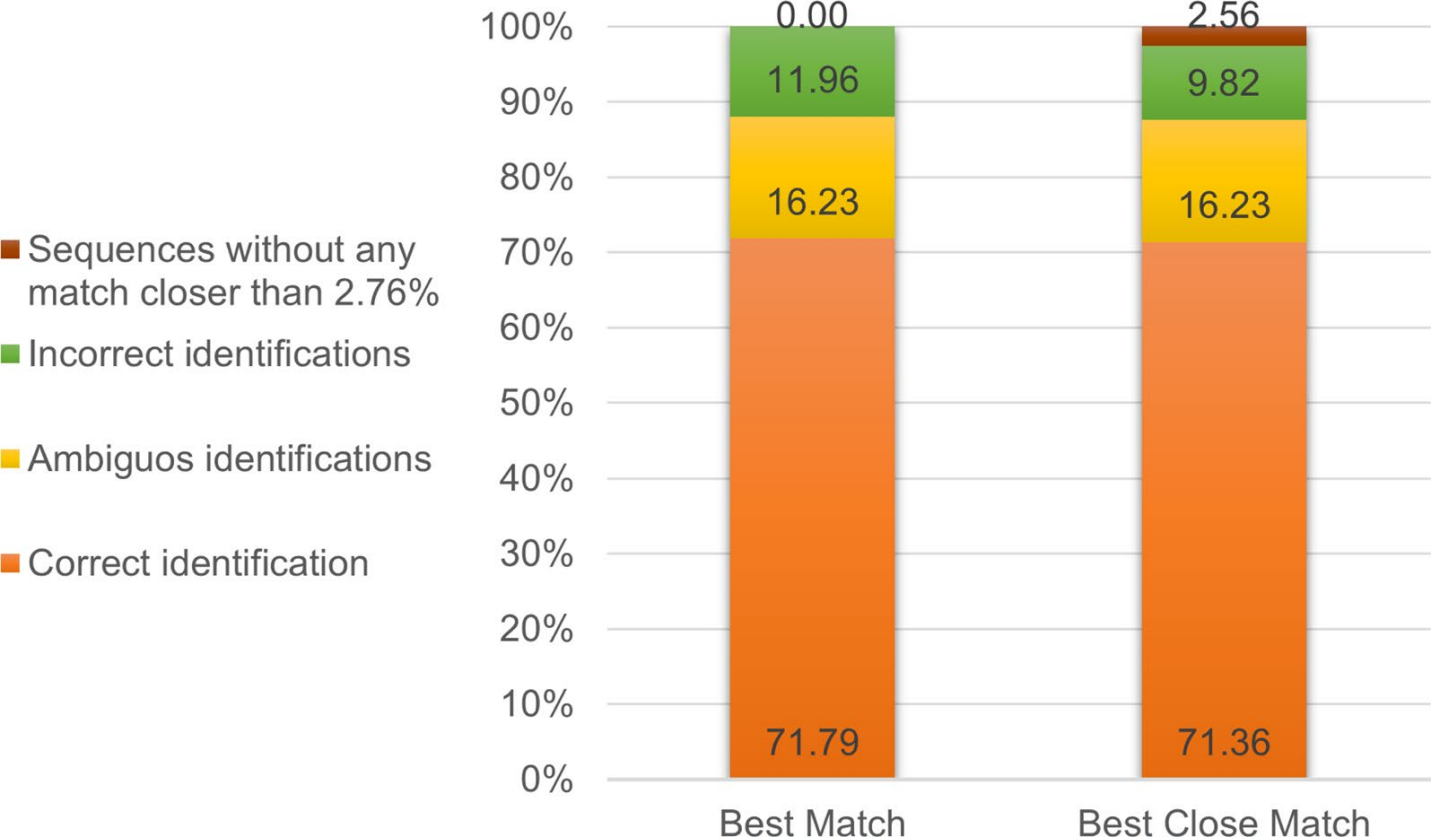
Efficacy of using COI barcodes for species identification of black flies, based on "Best Match" and "Best Close Match", expressed as percentages

The identification success rates for the 234 COI barcodes, based on "Best Match" and “Best Close Match” criteria, were 71.79% and 71.36%, respectively; ambiguous identification rates were 16.23%, and incorrect identification rates were 11.96% and 9.82%, respectively (Fig. 1). The percentage of sequences without any match closer than 2.76% for the "Best Close Match" criteria was 2.56%.

**Fig. 2:**
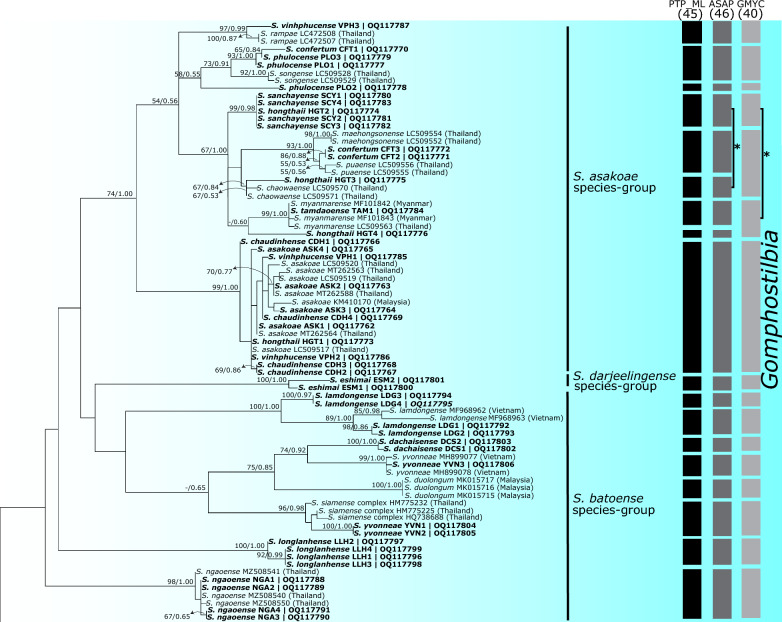
Maximum likelihood tree for the clade of the subgenus *Gomphostilbia* based on COI sequences. Bootstrap/posterior probabilities are shown as [ML/BI] on or near the branches. Values < 50/0.50 are not shown. The scale bar represents 0.05 substitutions per nucleotide position. Three columns on the right show OTUs delimited by PTP_ML, ASAP, and GMYC delimitation analyses. Asterisks (*) with a line joining entities indicate that the taxa were identified as one OTU by ASAP and GMYC methods, respectively

**Fig. 3:**
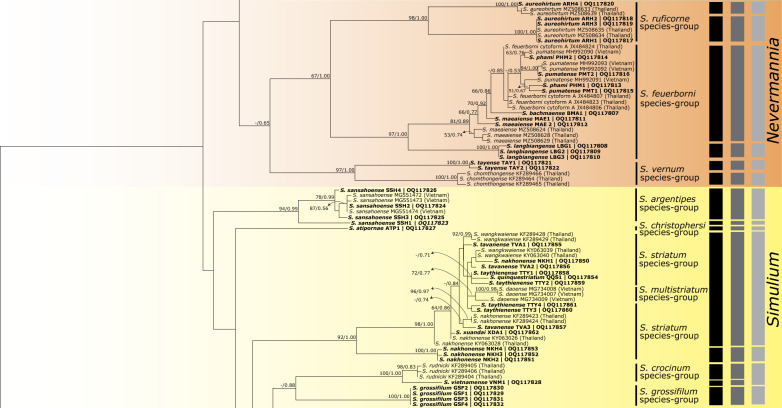
Continued maximum likelihood tree for the clades of the subgenera *Nevermannia* and *Simulium* based on COI sequences. Bootstrap/posterior probabilities are shown as [ML/BI] on or near the branches. Values < 50/0.50 are not shown. The scale bar represents 0.05 substitutions per nucleotide position. Three columns on the right show OTUs delimited by PTP_ML, ASAP, and GMYC delimitation analyses

**Fig. 4:**
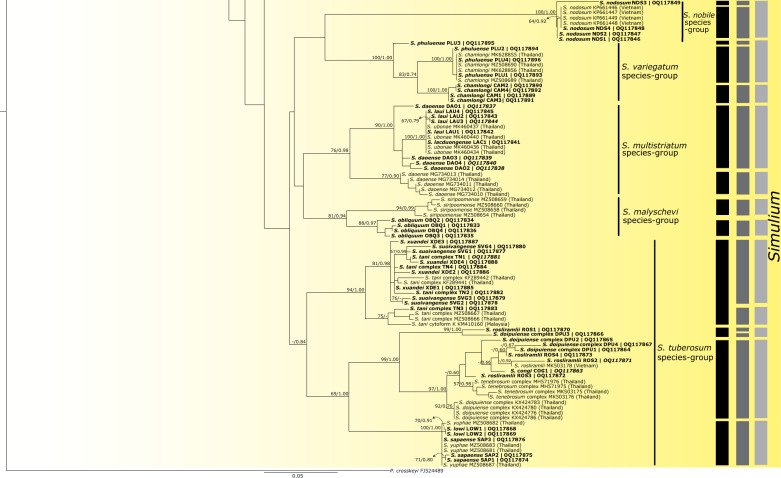
Continued maximum likelihood tree for the clade of the subgenus *Simulium* based on COI sequences. Bootstrap/posterior probabilities are shown as [ML/BI] on/near the branches. Values < 50/0.50 are not shown. The scale bar represents 0.05 substitutions per nucleotide position. Three columns on the right show OTUs delimited by PTP_ML, ASAP, and GMYC delimitation analyses

**Fig. 5:**
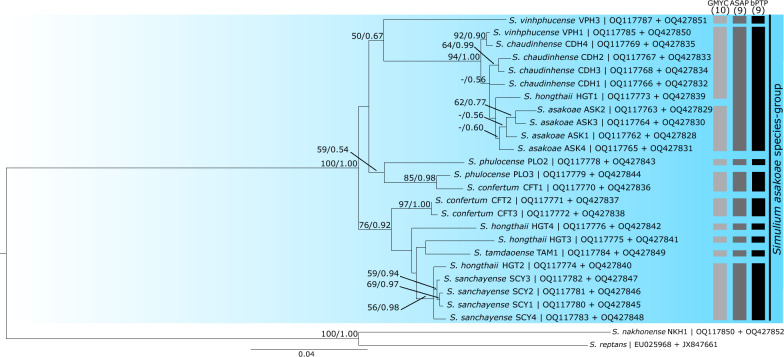
Maximum likelihood tree of members of the *Simulium asakoae* species group in Vietnam, based on concatenated COI and BZF sequences. Bootstrap values of maximum likelihood and posterior probabilities of Bayesian inference are shown on the branches as [ML/BI]. Values < 0.50/50% are not shown. The scale bar represents 0.04 substitutions per nucleotide position. Three columns on the right show OTUs delimited by GMYC, ASAP, and bPTP analyses

Phylogenetic analyses using ML and BI methods revealed similar tree topologies for COI and concatenated datasets. Therefore, only the ML trees of each dataset are presented (Figs. 2, 3, 4 for the COI dataset; Fig. 5 for the concatenated dataset; BI trees are shown in Additional file 3: Fig. S1 and Additional file 4: Fig. S2), with supporting values [ML/BI] noted near the branches. The ML tree of the COI dataset recovered three main clades of the three subgenera, *Gomphostilbia*, *Nevermannia*, and *Simulium* (Figs. 2, 3, 4). Most species were grouped with their respective species groups, resulting in 13 of the 16 species groups recovered being monophyletic. The *Simulium batoense* species group was polyphyletic because its clade included members of the *S. asakoae* and *S. darjeelingense* species groups. Three specimens of *S. daoense* (accessions MG734007–MG734009) in the *S. multistriatum* species group clustered with the clade of the *S. striatum* species group, rendering the *S. multistriatum* species group polyphyletic and the *S. striatum* species group paraphyletic. Of 45 nominal species collected in Vietnam, 13 were recovered as monophyletic based on COI sequences. The counts for the monophyletic species excluded the eight species with single specimens, as these species cannot exhibit non-monophyly [67].

Of 61 nominal species, the number of OTUs revealed by each species' delimitation analysis was 45 by the PTP_ML method, 46 by the ASAP method, and 40 by the GMYC method (Figs. 2, 3, 4). PTP_ML, ASAP, and GMYC methods delimited 32 OTUs that shared the same assignation. On the other hand, GMYC, ASAP, and bPTP methods yielded similar results for the concatenated dataset for the *S. asakoae* species group and delimited 10, nine, and nine OTUs, respectively (Fig. 5). Both ASAP and bPTP were congruent in delimiting the same nine OTUs.

Among the 14 nominal species of the subgenus *Gomphostilbia* in Vietnam (Fig. 2), *Simulium dachaisense*, *S. eshimai*, *S. lamdongense*, *S. longlanhense*, and *S. ngaoense* were recovered as monophyletic based on COI sequences. The species status of these morphologically defined taxa was supported by the molecular delimitations of PTP_ML, ASAP, and GMYC, except *S. lamdongense*, which had two distinct OTUs. *Simulium yvonneae* was recovered as polyphyletic, as the individuals were split into two distinct well-supported lineages. In the second lineage, two specimens of *S. yvonneae* (accessions OQ117804 and OQ117805) formed a sister clade with the *Simulium siamense* complex from Thailand, and together they were delimited as a distinct OTU. All eight nominal species of the *S. asakoae* species group in Vietnam were recovered as non-monophyletic based on COI sequences, with incongruities between the molecular delimitations of PTP_ML, ASAP, and GMYC and the morphological delineations. On the other hand, based on the concatenated dataset, *S. asakoae* s. str. was recovered as monophyletic despite weak support (Fig. 5), and its species status was supported by the GMYC method.

Among the seven nominal species of the subgenus *Nevermannia* in Vietnam (Fig. 3), *Simulium langbiangense*, *S. aureohirtum*, and *S. tayense* were recovered as monophyletic based on COI sequences. The molecular delimitations by PTP_ML, ASAP, and GMYC conformed to the morphological delineations of *S. langbiangense* and *S. tayense*. *Simulium aureohirtum*, however, was delimited as two separate OTUs. Contrarily, *Simulium phami*, *S. pumatense*, and *S. maeaiense* were recovered as paraphyletic based on COI sequences. *Simulium bachmaense* formed a sister clade with *S. phami*, *S. pumatense*, and *S. feuerborni* cytomform A. Together with *S. maeaiense*, these five nominal species were delimited as a single OTU by PTP_ML, ASAP, and GMYC.

Of the 24 nominal species of the subgenus *Simulium* sampled in Vietnam (Figs. 3, 4), *Simulium sansahoense*, *S. obliquum*, *S. lowi*, *S. grossifilum*, and *S. nodosum* were recovered as monophyletic based on COI sequences. The molecular delimitations by PTP_ML, ASAP, and GMYC agreed with the morphological delineations of *S. obliquum* and *S. grossifilum*. *Simulium sansahoense* and *S. nodosum* were delimited as two OTUs by all the species delimitation methods and by PTP_ML and ASAP, respectively. *Simulium chamlongi* from Vietnam formed a distinct clade and was delimited as a discrete OTU by all the molecular delimitation analyses. Conversely, *Simulium phuluense* (except one specimen with accession OQ117895) and *S. chamlongi* from Thailand were merged into a single OTU by PTP_ML, ASAP, and GMYC. Non-monophyly was observed in all nominal species of the *S. multistriatum*, *S. striatum*, and *S. tuberosum* (except *S. lowi*) species groups in Vietnam. Incongruities also manifested between the molecular delimitations and the morphological delineations for the nominal species in these groups.

## Discussion

The overall efficacy of identification of nominal species was 71% for the 234 COI barcodes of black flies, based on "Best Match" and "Best Close Match" methods. Ambiguous and incorrect identifications were observed for closely related species and members of species complexes, particularly in the *S. asakoae*, *S. feuerborni*, *S. multistriatum*, *S. striatum*, *S. tuberosum*, and *S. variegatum* species groups. DNA barcoding for identifying species depends on the gap between intra- and interspecific genetic divergence [68]. The degree of overlap of intra- and interspecific distances tends to be more significant when a large proportion of closely related species and members of species complexes are involved [48, 69, 70]; thus, the rather low success rate of identification in the present study was not unexpected. Ambiguous and incorrect identifications were associated with species recovered as non-monophyletic in the phylogenetic analyses.

Based on COI sequences, 13 of the 45 species (excluding those based on single specimens) sampled in Vietnam were unambiguously identified in the phylogenetic analyses. The COI gene provided insufficient resolution for differentiating most members of the *S. asakoae*, *S. feuerborni*, *S. multistriatum*, *S. striatum*, *S. tuberosum*, and *S. variegatum* species groups. The clustering of closely related nominal species in these species groups corresponds with a low level of minimum interspecific genetic distance between them, impeding the efficacy of the COI gene for identification. Previous studies revealed no or very low levels of minimum interspecific genetic divergence in some putative species of black flies, particularly closely related species [24, 28, 29, 31, 71] and members of species complexes [21, 72].

In the *S. asakoae* species group of the subgenus *Gomphostilbia*, low minimum interspecific genetic distance was found between the following species pairs: *Simulium asakoae* s. str. and *S. chaudinhense* (0.25%), *S. confertum* and *S. phulocense* (0.99%), and *S. hongthaii* and *S. sanchayense* (0.00%). The implication is that these pairs are genetically closely related and morphologically similar [3, 6, 7]. Nevertheless, these pairs can be morphologically distinguished from their sister species based on the number of male upper-eye facets and differences in pupae [3, 6, 7]. In the *S. asakoae* species group in Thailand where there is considerable morphological variation but few COI gene changes, the inference is that some members of the group might have recently diverged from a common ancestor, rendering the use of the COI gene insufficient to discriminate the species [31]. Previous studies have demonstrated limited differentiation among some members of the *S. asakoae* species group when using only the COI gene [29, 34].

Alternatively, some morphologically defined species in Vietnam might be based on intraspecific variation. For instance, *Simulium huense* (previously as *S. cavum*) in Vietnam is morphologically only marginally different from *S. yuphae* cytoform A in Thailand, based on the number of male upper-eye facets and the size of the tubercles on the pupal frons [6]. A cytogenetic study found no chromosomal differences between the Vietnamese and Thai samples, suggesting either that the two nominal species are homosequential (i.e. morphologically slightly distinct species with identical banding sequences of polytene chromosomes [73, 74]) or that *S. huense* is conspecific with *S. yuphae* cytoform A, in which case the morphological differences correspond to intraspecific variation, rendering *S. huense* a synonym of *S. yuphae* cytoform A [20]. Given the significant geographic separation between the two nominal species, the latter interpretation might have more weight.

Based on our preliminary results for discriminating the members of the *S. asakoae* species group, the tree topology of the COI dataset differs from that of the BZF dataset, in which no species are monophyletic based on the COI dataset (Additional file 5: Fig. S3). In contrast, the BZF dataset shows monophyly of *S. asakoae* s. str., with very low bootstrap support; further investigation is required to resolve the relationships of the other seven sampled species in the group (Additional file 6: Fig. S4). Given that combining nuclear and mitochondrial genes can improve the resolution of species boundaries [21, 34], we concatenated mitochondria-encoded COI and nuclear-encoded BZF sequences to resolve members of the *S. asakoae* species group in Vietnam. Consequently, *S. asakoae* s. str. was successfully distinguished from the other group members, with improvements in the clustering of the other seven species (Fig. 5) compared with the BZF dataset alone. Our COI and BZF sequences of *S. asakoae* s. str., therefore, could facilitate the identification of this species when only females are collected, as in a previous study [32]. The current study reveals the limited resolving power of the BZF gene in differentiating Vietnamese members of the *S. asakoae* species group (excepting *S. asakoae* s. str.), suggesting that the genetic divergence for this marker might be insufficient to differentiate these closely related, morphologically similar species despite its value in previous studies [35, 36, 38, 39]. BZF and ECP1 genes have limited ability to discriminate Nearctic members of the *Simulium fibrinflatum* and *S. taxodium* subgroups in the *S. jenningsi* species group and Thai members of the *S. striatum* species group, respectively [37, 75]. The resolving power of the ECP1 gene for Vietnamese members of the *S. asakoae* species group has yet to be evaluated, as the samples from this study were not successfully amplified by the primer pair from [21]. Perhaps, apart from the BZF and ECP1 genes, other more variable molecular markers, such as the 5-intron gene (5intG) [37] or molybdenum cofactor sulfurase (MCS) [76], would be helpful in delineating closely related species of the *S. asakoae* species group.

In the *S. feuerborni* species group of the subgenus *Nevermannia*, *S. pumatense* is paraphyletic with the inclusion of *S. phami*. In the absence of *S. phami*, *S. pumatense* forms a distinct clade [8]. Our study reveals an unexpected sister relationship between *S. phami* and *S. pumatense*, with a minimum interspecific genetic divergence of 0.50%, although both species are morphologically distinguishable from each other based on the number of primary rays in the larval labral fan, the number of pupal gill filaments, the number of male upper-eye facets, and the number of conical processes in the female cibarium [5, 8]. We note, however, that certain morphological characters, such as the number of rays in the primary labral fan, are subject to environmental influence [77].

In the *S. multistriatum* and *S. striatum* species groups of the subgenus *Simulium*, the COI gene was ineffective in differentiating the species, agreeing with previous studies attempting to resolve species boundaries of Thai members in both species groups [23, 28, 38, 75]. Members of the *S. multistriatum* and *S. striatum* species groups can be differentiated by the ECP1 and BZF genes, respectively [23, 35, 36, 39], indicating inadequate phylogenetic signal of COI sequences. In the *Simulium multistriatum* species group, *S. lacduongense*, *S. laui*, and *S. ubonae* are closely related, with interspecific genetic distances of 0.00–0.25%, corroborating the shared morphological characteristics [78]. In the female, *S. lacduongense* is characterized by having a haired basal radial wing vein, whereas the vein is bare in *S. laui* and *S. ubonae*; *S. laui* is distinguished from *S. ubonae* by having posteriorly divergent inner margins of the ovipositor valves (slightly concave in *S. ubonae*) [6, 7, 78]. These three species are distinguished based on the number of male upper-eye facets [6, 7, 78]. In the *S. striatum* species group, the females of *Simulium tavanense*, *S. taythienense*, *S. quinquestriatum*, and *S. nakhonense* are morphologically similar [4, 7, 79, 80], but are morphologically distinguishable in the male and pupa. *Simulium tavanense* is distinguished from the other three species by having a haired basal portion of the radial wing vein in males; *S. taythienense* is distinguished from *S. tavanense* and *S. quinquestriatum* by having short brassy hairs on the male scutum and differs from *S. nakhonense* based on the number of male upper-eye facets; *S. quinquestriatum* is distinguished from *S. nakhonense* based on the pupal gill arrangement [4, 7, 79, 80].

In the *S. tuberosum* species group of the subgenus *Simulium*, *S. xuandei*, *S. suoivangense*, and the *S. tani* complex are clustered in a clade. This result is not surprising, as *S. xuandei* and *S. suoivangense* are members of the *S. tani* complex [6, 7, 21]. Current findings corroborate those of [21] who reported limited utility of the COI gene in resolving members of the *S. tani* complex. However, members of the *S. tani* complex can be distinguished using the ECP1 gene [21], implying inadequate phylogenetic signal of COI sequences. Additionally, the species status of *S. xuandei* and *S. suoivangense* was cytogenetically supported [20]. Adults of *S. xuandei*, *S. suoivangense*, and the *S. tani* complex are morphologically similar, although *S. xuandei* can be differentiated based on the divergence angle in the pupal gills [6, 7]. The latter two species can be distinguished by the pupal terminal hooks, which are present in the *S. tani* complex but absent in *S. souivangense* [7]. In the second clade of the *S. tuberosum* species group, the genetically close relationship (minimum interspecific genetic distance = 0.74%) between the *S. doipuiense* complex and *S. rosliramlii* is supported chromosomally by the sharing of a chromosomal rearrangement by *S. rosliramlii* (as *S. rufibasis* cytoform B) and *S. doipuiense* cytoform A collected from the same site (Sapa, Lao Cai Province, Vietnam); the occasional presence of the shared chromosome character is possibly the result of introgressive hybridization or incomplete lineage sorting [20]. Nevertheless, several morphological features can help differentiate *S. rosliramlii* and the *S. doipuiense* complex, such as the colour of the female antennae, the number of male upper-eye facets, and the presence of tubercles on the pupal frons [7, 81].

In the *S. variegatum* species group of the subgenus *Simulium*, three individuals of *S. phuluense* (accessions OQ117893, OQ117894, and OQ117896) cluster with *S. chamlongi* from Thailand, with very low minimum interspecific genetic divergence (0.00–0.25%), rendering both species non-monophyletic. Current findings are comparable to those by [82], who showed that *S. chamlongi* from Thailand is not monophyletic. The specimens of *S. phuluense* analysed were topotypes, whereas the sequences of *S. chamlongi* from Thailand that were retrieved from the NCBI GenBank were > 100 km from the type locality of *S. chamlongi* (Doi Inthanon, Chiang Mai Province, Thailand). Thus, both taxa might be conspecific or molecularly inseparable based on the genes examined. *Simulium phuluense* can be morphologically distinguished from *S. chamlongi* based on the number of teeth on the female mandible, the number of male upper-eye facets, the presence of spine-combs on pupal abdominal segment 9, and the number of primary rays in the larval labral fan [7, 80]. Morphological re-examination and cytogenetic study are needed for taxonomic resolution.

Despite the limited resolving power of the COI gene in differentiating closely related, morphologically similar species of black flies, mainly due to lower genetic distances among them, it remains the genetic marker of choice for black flies in studies of DNA barcoding [26, 28, 29]. The lack of monophyly among closely related species in the six species groups that we evaluated in Vietnam could be due to inadequate phylogenetic signal for the DNA sequences used, incomplete lineage sorting, conspecificity, and introgression [83]. When the rate of speciation exceeds the rate of gene evolution, fewer phylogenetically informative characters exist between species, and even mitochondrial genes, which typically evolve faster than certain nuclear genes, may not have sufficient variation to distinguish recently diverged species [83]. Weak support (< 50%/0.50) was recovered for the majority of the nodes within the non-monophyletic clades of *S. asakoae* + *S. chaudinhense*, *S. phami* + *S. pumatense*, *S. tavanense* + *S. taythienense* + *S. quinquestriatum* + *S. nakhonense*, *S. suoivangense* + the *S. tani* complex + *S. xuandei*, the *S. doipuiense* complex + *S. rosliramlii*, and *S. chamlongi* + *S. phuluense*, as well as in the phylogenetic trees of the concatenated COI and BZF dataset. The weak support indicates that the COI sequences and the COI + BZF sequences, respectively, in this study might provide insufficient variation to resolve these closely related, or possibly recently diverged [72], or conspecific taxa. In some instances, however, non-monophyly in the clades of *S. confertum* + *S. phulocense*, *S. hongthaii* + *S. sanchayense*, and *S. lacduongense* + *S. laui* + *S. ubonae* was strongly supported (> 70%/0.70), which might reflect true non-monophyly at the species level, although we cannot rule out the possibility of inadequate phylogenetic signal [84]. Incomplete lineage sorting could impact mitochondrial phylogenies, especially in rapidly radiating taxa, where the taxa undergo speciation events before the sorting of allelic lineages is complete [83]. Low genetic distances among closely related species in this study support the incomplete lineage sorting hypothesis [21, 84]. Introgressive hybridization is characterized by interspecific mating followed by backcrossing, leading to polyphyly by introducing phylogenetically divergent allelic lineages across species boundaries [83]. Some taxa of the *S. doipuiense* complex and *S. rosliramlii* at the same site were reported to share a chromosomal rearrangement (IIIL-64), possibly due to incomplete lineage sorting or introgressive hybridization [20]. The possibility of introgression causing non-monophyly in both species cannot be precluded, as separating the two causes of species-level non-monophyly is challenging [83]. Further cytogenetic investigations are required based on more sample collections, particularly where both species co-exist. In cases where the COI gene is ineffective, more variable molecular markers such as BZF or ECP1 are encouraged for use in future studies to sort out the interspecific relationships of members of the *S. feuerborni*, *S. multistriatum*, *S. striatum*, *S. tuberosum*, and *S. variegatum* species groups. Future population genetic studies are needed to investigate the presence of shared identical sequences among closely related species of Vietnamese black flies.

In our study, 15 species of black flies have high levels of intraspecific genetic divergence (> 3%), associated with their broader geographic distributions in Vietnam (Table 2). For example, a paratype of *S. confertum* from Lam Dong Province in southern Vietnam and two specimens from Lao Cai Province in northern Vietnam had a genetic divergence of 5.45%. Four specimens of *S. hongthaii* from four locations in three provinces in Vietnam had intraspecific genetic variation of 3.47–8.66%, implying that *S. hongthaii* might be a species complex. A paratype of *S. vinhphucense* from Vinh Phuc Province in northern Vietnam was genetically divergent (maximum = 7.92%) from two specimens from Nghe An Province in the northern part of central Vietnam. Likewise, two paratypes of *S. lamdongense* from Lam Dong Province in southern Vietnam had a maximum genetic divergence of 4.70% from two specimens in Nghe An Province in the northern part of central Vietnam.

*Simulium aureohirtum* exhibits a high level of intraspecific genetic variation (maximum = 8.42%) comparable to values in previous studies [28, 29] and forms two supported lineages, in accordance with a previous phylogeographic study based on mitochondrial cytochrome c oxidase II (COII) sequences [85]. However, no evidence of chromosomally defined species has been found in *S. aureohirtum* from Thailand [86]. A cytogenetic study is needed for populations in Vietnam.

The greatest intraspecific genetic variation (15.10%) was between paratypes of *S. daoense* in this study (accessions OQ117837–OQ117840) and topotypes of *S. daoense* (accessions MG734007–MG734009) [87]. The following nominal species yielded high levels of intraspecific genetic variation, although the analysed specimens are paratypes and topotypes: *S. phulocense* (maximum = 5.20%), *S. yvonneae* (maximum = 9.41%), *S. sansahoense* (maximum = 4.95%), *S. rosliramlii* (maximum = 8.17%), and *S. phuluense* (maximum = 4.46%). A 3% genetic divergence is a common cut-off value in delimiting species boundaries of black flies [12, 21, 32]. These high levels of intraspecific variation should prompt investigations for cryptic species. Apart from possessing high intraspecific genetic variation, the specimens of these species were molecularly delimited into two distinct OTUs and formed deeply divergent lineages. Discovering cryptic species in black fly species collected from type localities is not uncommon [88, 89]. The *S. doipuiense* and *S. tani* complexes, which consist of multiple taxa [90], have a maximum intraspecific genetic variation of 7.92% and 5.94%, respectively, which is comparable to values previously reported [29], as well as values for other complexes of black flies [26]. High levels of intraspecific genetic variation in both species complexes correspond with a cytogenetic study that revealed five cytoforms in the *S. doipuiense* complex and four cytoforms in the *S. tani* complex in Vietnam [20].

DNA barcoding relies on a comprehensive reference library to assign unknown specimens to known species [91]. Of 48 novel black fly species discovered in Vietnam, with only COI sequences of seven species previously available in GenBank, our study contributed additional COI sequences of 27 species, with the intent of constructing a comprehensive reference library for black flies in Vietnam to facilitate rapid species identification, particularly of potential pests and vectors, despite an overall 71% barcoding success. Phylogenetic analyses in our study revealed that most black fly taxa align with their respective species group, providing strong support for their assignment based on morphological features. By analysing the COI sequences of Vietnamese black flies, we discovered divergent lineages and potential cryptic species in 13 taxa, as well as in two additional taxa (the *S. doipuiense* and *S. tani* complexes), in accordance with previous cytogenetic findings [20]. Unambiguous identification of *S. asakoae* s. str. demonstrates the addition of a fast-evolving nuclear gene that could be more informative in resolving closely related species. Our results provide baseline data for future investigations that should employ an integrated approach in resolving problematic black fly taxa. The results also provide a foundation for future research on the biological aspects of black flies, particularly the biting behaviour of female black flies in Vietnam. The discovery of 73 black fly species in only five of the 58 provinces in Vietnam also encourages future studies to extend taxon sampling across the country. We suggest that novel species will be found in the unexplored provinces, supporting the status of Vietnam as a biodiversity hotspot. Given the rarity of habitat-specialised species of black flies, there is a need to speed their discovery before it is too late, as their habitats might be threatened by the increasing human population in Vietnam [20].

Although classic DNA barcoding can be useful for species identification and discovery of cryptic species, it is not a test of biological species in the same way that polytene chromosome analyses, at least in sympatric situations, can be used to infer reproductive isolation. We, therefore, support an integrated approach to the taxonomy of black flies, involving the concomitant use of morphology, chromosomes, DNA, and ecology. An effective but time-consuming approach is to use the same individual (larva) to extract morphological, chromosomal, and molecular information. By using specimens from the type localities of most of the species in the present study, we have identified a number of taxonomic problems through barcoding analyses for which this integrated approach can now be brought to bear.

## Conclusions

This study represents the first comprehensive molecular study of species delineation and evolutionary relationships of black flies in Vietnam, revealing that DNA barcoding using the COI gene is helpful but not entirely satisfactory (71%) for identifying 45 species. DNA barcoding effectively differentiates most taxonomically well-defined species and helps reveal cryptic diversity. Conversely, it is ineffective in discriminating closely related, morphologically similar nominal species and members of species complexes in the *S. asakoae*, *S. feuerborni*, *S. multistriatum*, *S. striatum*, *S. tuberosum*, and *S. variegatum* species groups, possibly because of factors such as inadequate phylogenetic signal of the DNA sequence used, incomplete lineage sorting, conspecificity, and introgressive hybridization. Based on concatenated COI and BZF sequences, only *S. asakoae* s. str. in the *S. asakoae* species group can be identified, suggesting the need for more variable molecular markers such as the 5intG or MCS gene. Nonetheless, BZF or ECP1 can be used for species resolution of the members of the other five species groups. An integrated morphological, cytogenetic, ecological, and molecular approach would further benefit the systematics of black flies in Vietnam.

### Supplementary Information

**Additional file 1: Table S1.** Interspecific genetic distances of 61 species (COI) based on uncorrected *p*-distance expressed as percentages.

**Additional file 2: Table S2.** Intra- and interspecific genetic distances of eight members in the *S. asakoae* species-group (COI + BZF) based on uncorrected *p*-distance expressed as percentages.

**Additional file 3: Figure S1.** Bayesian inference tree for *Simulium* black flies based on COI sequences.

**Additional file 4: Figure S2.** Bayesian inference tree for members of the *S. asakoae* species-group based on concatenated COI and BZF sequences.

**Additional file 5: Figure S3.** Maximum likelihood tree for members of the *S. asakoae* species-group based on COI sequences.

**Additional file 6: Figure S4.** Maximum likelihood tree for members of the *S. asakoae* species-group based on BZF sequences.

## Data Availability

All data generated or analysed during this study are included in this published article and its additional files. The newly generated sequences were deposited in the GenBank database under the accession numbers OQ117762–OQ117896 (COI gene) and OQ427828–OQ427851 (BZF gene). The relevant information for species of black flies in Vietnam is also available at https://www.gbif.org/dataset/c80987f7-f87a-4ae3-a2cc-ccd59bc951e8.
