# Supplementary figures and images for: From bites to barcodes: uncovering the hidden diversity of black flies (Diptera: Simuliidae) in Vietnam
Source: Parasit Vectors. 2023 Aug 7;16:266. doi: 10.1186/s13071-023-05892-0 (PMC10405495; doi:10.1186/s13071-023-05892-0)

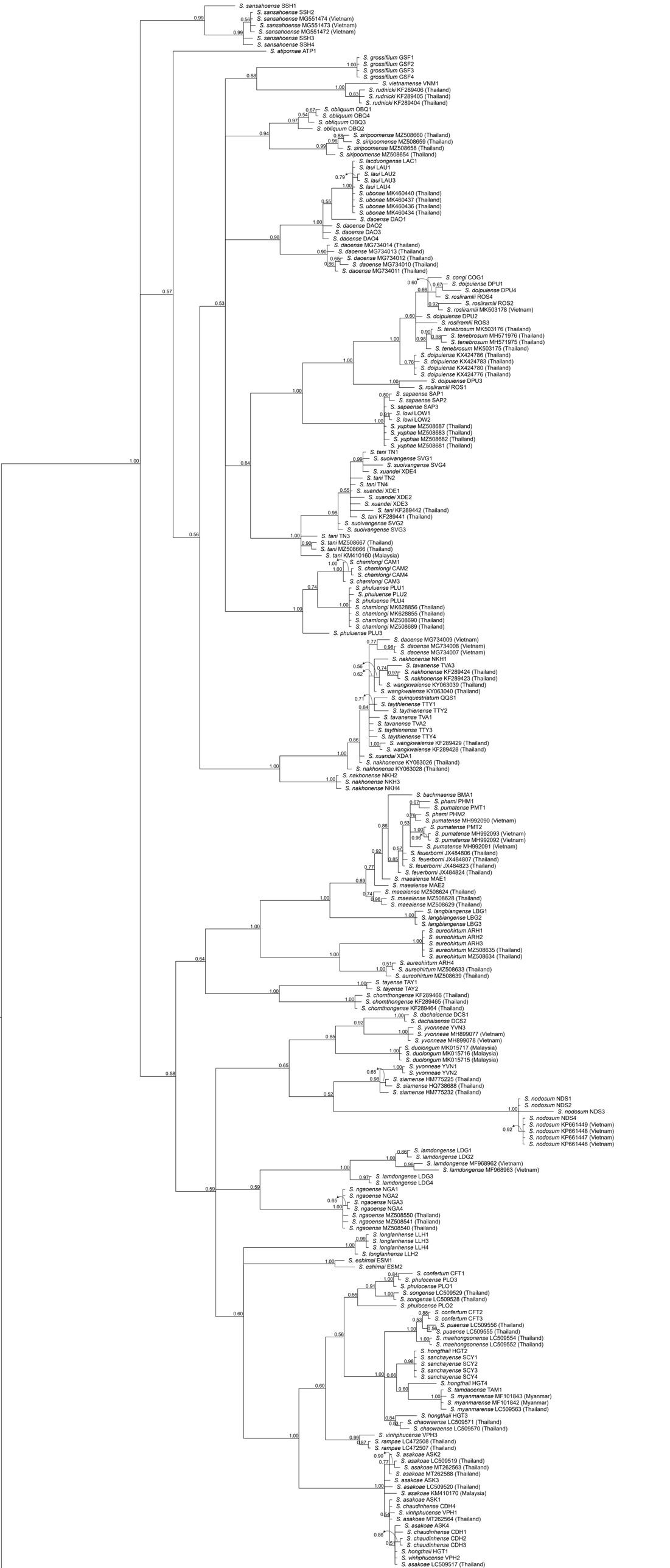

*P. crosskeyi* FJ524489

0.05

Supplement: Supplementary file 3 — Additional file 3: Figure S1. Bayesian inference tree for Simulium black flies based on COI sequences. [file 13071_2023_5892_MOESM3_ESM.pdf]

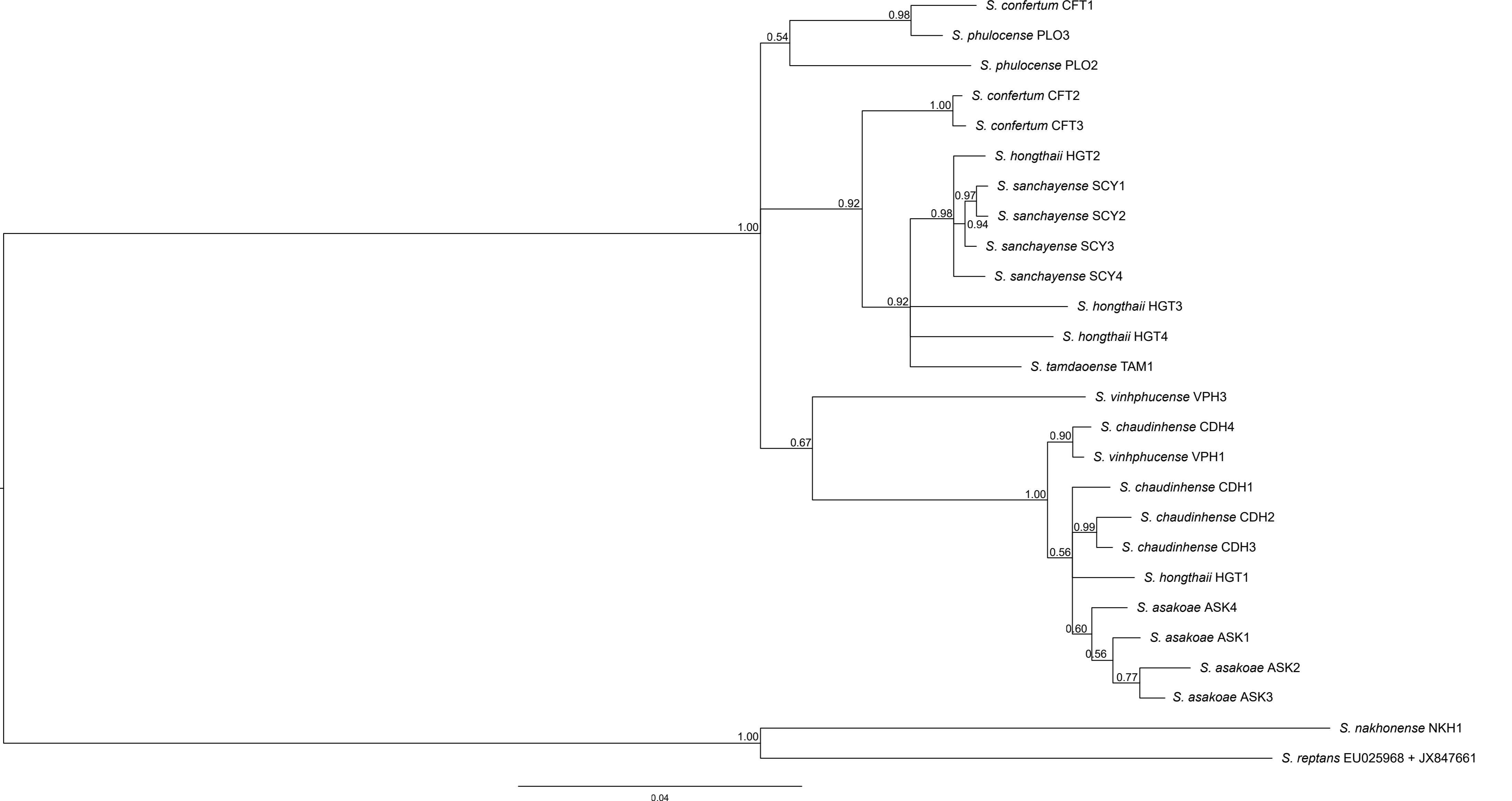

Supplement: Supplementary file 4 — Additional file 4: Figure S2. Bayesian inference tree for members of the S. asakoae species-group based on concatenated COI and BZF sequences. [file 13071_2023_5892_MOESM4_ESM.pdf]

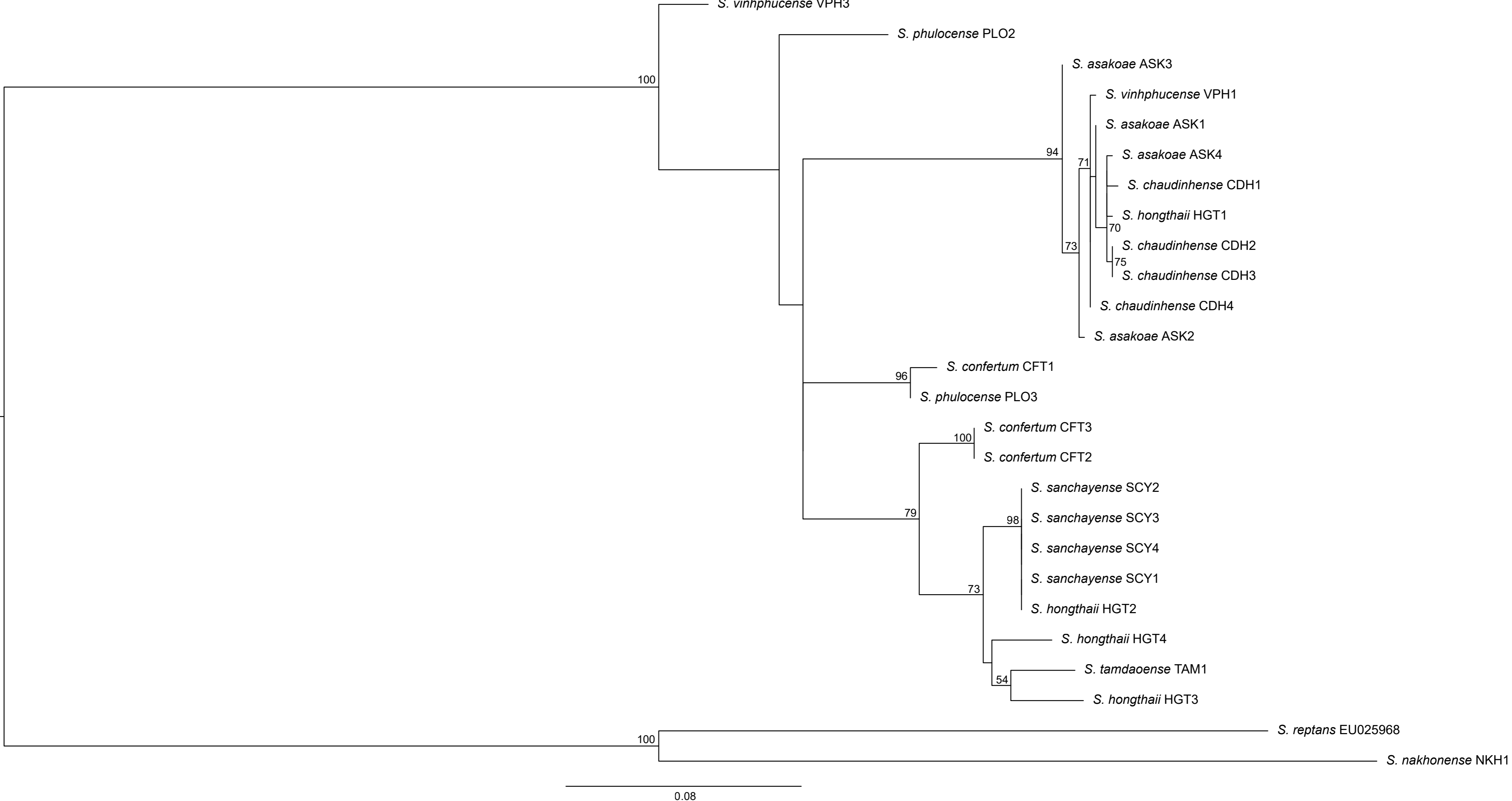

Supplement: Supplementary file 5 — Additional file 5: Figure S3. Maximum likelihood tree for members of the S. asakoae species-group based on COI sequences. [file 13071_2023_5892_MOESM5_ESM.pdf]

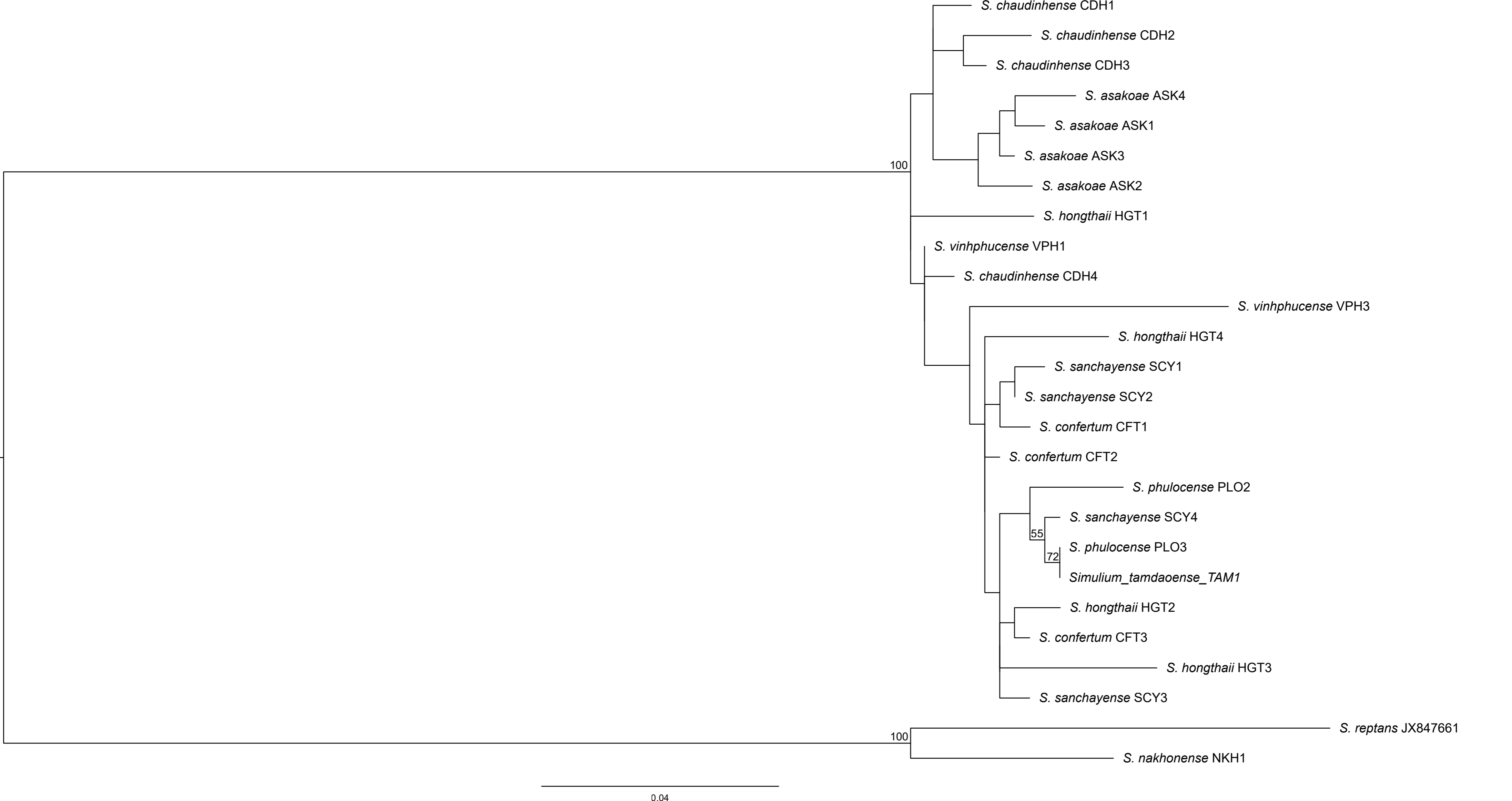

Supplement: Supplementary file 6 — Additional file 6: Figure S4. Maximum likelihood tree for members of the S. asakoae species-group based on BZF sequences. [file 13071_2023_5892_MOESM6_ESM.pdf]
